# Imputation for sequencing variants preselected to a customized low-density chip

**DOI:** 10.1038/s41598-020-66523-7

**Published:** 2020-06-12

**Authors:** Aoxing Liu, Mogens Sandø Lund, Didier Boichard, Xiaowei Mao, Emre Karaman, Sebastien Fritz, Gert Pedersen Aamand, Yachun Wang, Guosheng Su

**Affiliations:** 1 Center for Quantitative Genetics and Genomics, Aarhus University, 8830 Tjele, Denmark; 2 Key Laboratory of Animal Genetics, Breeding and Reproduction, MARA; National Engineering Laboratory for Animal Breeding, College of Animal Science and Technology, China Agricultural University, 100193 Beijing, P.R. China; 3 GABI, INRA, AgroParisTech, Université Paris Saclay, 78350 Jouy-en-Josas, France; 4 Key Laboratory of Vertebrate Evolution and Human Origins, Institute of Vertebrate Paleontology and Paleoanthropology, Chinese Academy of Sciences, 100044 Beijing, P.R. China; 5 CAS Center for Excellence in Life and Paleoenvironment, 100044 Beijing, P.R. China; 6 ALLICE, 75012 Paris, France; 7 Nordic Cattle Genetic Evaluation, 8200 Aarhus, Denmark

**Keywords:** Animal breeding, Genotype

## Abstract

The sequencing variants preselected from association analyses and bioinformatics analyses could improve genomic prediction. In this study, the imputation of sequencing SNPs preselected from major dairy breeds in Denmark-Finland-Sweden (DFS) and France (FRA) was investigated for both contemporary animals and old bulls in Danish Jersey. For contemporary animals, a two-step imputation which first imputed to 54 K and then to 54 K + DFS + FRA SNPs achieved highest accuracy. Correlations between observed and imputed genotypes were 91.6% for DFS SNPs and 87.6% for FRA SNPs, while concordance rates were 96.6% for DFS SNPs and 93.5% for FRA SNPs. The SNPs with lower minor allele frequency (MAF) tended to have lower correlations but higher concordance rates. For old bulls, imputation for DFS and FRA SNPs were relatively accurate even for bulls without progenies (correlations higher than 97.2% and concordance rates higher than 98.4%). For contemporary animals, given limited imputation accuracy of preselected sequencing SNPs especially for SNPs with low MAF, it would be a good strategy to directly genotype preselected sequencing SNPs with a customized SNP chip. For old bulls, given high imputation accuracy for preselected sequencing SNPs with all MAF ranges, it would be unnecessary to re-genotype preselected sequencing SNPs.

## Introduction

In dairy cattle, with the availability of whole-genome sequencing (WGS) data (~ 27 million variants), a large number of causative loci or single nucleotide polymorphisms (SNPs) tightly linked to causative loci have been detected through association analyses^1,2^ and bioinformatics analyses^3,4^. The integration of these preselected sequencing SNPs into the genotype data of the standard SNP chip is expected to improve genomic prediction, which has been well documented in various studies^5–7^. On the one hand, compared with using the standard SNP chip, integrating preselected sequencing SNPs could better capture the information of causative loci instead of relying on the extensive linkage disequilibrium. On the other hand, compared with using (imputed) WGS SNPs, integrating only preselected sequencing SNPs could reduce the computational burden and avoid the noise originating from the inclusion of a large number of non-causative loci^8^. Besides, using sequencing SNPs preselected from bioinformatics analyses could benefit association studies. For example, in three French dairy breeds including Holsteins, Montbéliarde, and Normande, a set of sequencing SNPs pre-selected from functional annotations were confirmed to be significant associations for milk production, fertility, and embryo mortality in both within-breed association analyses and across-breed meta-analyses^9^.

No matter genomic prediction or association studies, a sufficiently large population with genotypes of preselected sequencing SNPs is essential in order to get benefits from preselected sequencing SNPs. Although the costs of WGS keep decreasing, obtaining genotypes of preselected sequencing SNPs by directly sequencing a large number of animals remains economically infeasible. An alternative is to use a customized SNP chip which can include the SNPs defined by customers. Under the project of EuroGenomics, a customized low-density chip^10^ was designed to combine SNPs of the standard low-density chip^11^ together with thousands of additional SNPs preselected through association analyses and bioinformatics analyses in major dairy breeds of Denmark-Finland-Sweden (DFS) and France (FRA). A previous study in genotyped animals of Danish Jersey showed that the prediction reliability of milk and protein improved by including DFS and/or FRA SNPs^7^. In Denmark, this customized low-density chip has started to be used to genotype young bulls and heifers, which were used to be genotyped by the standard low-density chip. However, there are still many animals that have already been genotyped by the standard SNP chip, e.g., the 54 K chip or the standard low-density chip, and thus lack of information on preselected sequencing SNPs (DFS and FRA SNPs). Because these animals are older than the animals genotyped with the customized SNP chip and may have phenotypes, they are especially important to assess the effects of the preselected sequencing SNPs.

Missing genotypes of an individual can be imputed by using the information from the family or/and the population^12^. Many studies have been performed to impute SNPs from a lower density SNP panel to a higher density SNP panel, with the majority focusing on SNPs from the standard SNP chips in which SNPs were evenly distributed on the genome and had relatively high minor allele frequency (MAF)^11,13–15^. Unlike the SNPs in the standard SNP chip, the preselected sequencing SNPs are unevenly distributed on the genome and some of the SNPs could have low MAF^7,16^. Brøndum *et al*.^16^ showed that the correlation between imputed and observed genotypes from the high-density chip to WGS was 0.90, 0.89, and 0.87 for Nordic Holsteins, Danish Jersey, and Nordic Red, with a combined reference population of 116 Nordic Holsteins, 15 Danish Jersey, 23 Nordic Red, and 16 Brown Swiss. For WGS data, the relatively low imputation accuracy was also one of the reasons caused limited improvements in genomic prediction compared with using a high-density SNP chip^8^. Instead of using the WGS data as a reference panel, using a customized low-density chip could achieve a much larger reference population but with lower linkage disequilibrium between preselected sequencing SNPs and the other SNPs in the chip. By using a customized low-density SNP chip as a reference panel, Sanchez *et al*.^17^ imputed 194 candidate variants for milk protein and fatty acid composition in Montbéliarde cows and showed that the mean squared correlation between imputed and true genotypes was 91.6% for SNPs with MAF higher than 1%.

The imputation accuracy could be highly influenced by the MAF of SNPs^18,19^. Ma *et al*.^18^ showed that the lower the MAF, the higher the concordance rate but the lower the correlation between imputed and observed genotypes, when imputing from 3 K to 54 K or from 54 K to the high-density chip in a combined population of Swedish and Finnish Red Cattle, no matter which imputation algorithm was applied. For the imputation of preselected sequencing SNPs using a customized low-density chip as a reference panel, the reference population usually comprises young animals. This situation could be rare when imputing SNPs in the standard SNP chip or to WGS, where the animals with the target SNP panel are usually key animals such as ancestors^20^. The relationship between reference and target populations can largely impact the imputation accuracy^21^. Therefore, the imputation accuracy of preselected sequencing SNPs and the influence of MAF on the imputation accuracy could differ for different groups of animals.

The integration of preselected DFS and/or FRA SNPs covered by the EuroGenomics customized SNP chip could benefit genomic prediction^7^. In the present study, the primary objective was to investigate the imputation accuracy for DFS + FRA SNPs in both contemporary animals and old bulls, using the customized low-density chip as a reference panel. Besides, we investigated the pattern of imputation accuracy in relation to MAF for the preselected DFS + FRA SNPs.

## Methods

### Ethics statement

All data were recorded as part of the routine genomic evaluation, no animal handling or experiment was performed specially for the present study.

### Data

A total of 3,745 Danish Jersey bulls, 1,168 US Jersey bulls, and 28,678 Danish Jersey cows were available in the present study. The Danish Jersey bulls were mainly genotyped with the Illumina Bovine SNP50 chip (54 K, Illumina, Inc). The US Jersey bulls, which had close relationships with Danish Jersey^22^, and currently used in routine genomic evaluation of Danish Jersey, were genotyped with either the 54 K chip or the GeneSeek Genomic Profiler HD chip (HD, GeneSeek, Neogen Corp.). For US Jersey bulls, only SNPs in the 54 K chip were kept for analyses. Danish Jersey cows were genotyped with the 54 K chip (3%), the standard Illumina Bovine LD chip (standard LD, Illumina, Inc.) (49%), or the customized Illumina Bovine LD chip^10^ (customized LD, Illumina, Inc.) (48%).

The customized LD chip included SNPs in the standard LD chip, and more importantly, additional SNPs preselected from the WGS data in major dairy breeds of Denmark-Finland-Sweden (DFS) and France (FRA)^10^. For DFS SNPs, a total of 1,715 sequencing SNPs were preselected through association analyses based on imputed WGS data in Nordic Holsteins, Nordic Red, and Danish Jersey^5,7^. For FRA SNPs, a total of 4,325 SNPs were preselected through both association analyses and bioinformatics analyses^10^. In bioinformatics analyses, SNPs from regulatory regions of genes, breakpoints of structural variations, and SNPs with strong predicted effects using Variant Effect Predictor^23^ (e.g., frameshift, stop gain, splicing site, and non-synonymous substitution) were preselected^10^.

The SNPs on sex chromosome or with an unknown position, and with monomorphism or multiple alleles were removed from analyses using Plink^24^. Ultimately, 53,921 autosomal SNPs (including the SNPs of the 54 K chip and the preselected sequencing SNPs) from 33,591 individuals were available for further analyses.

### Genotype imputation

Genotype imputation was carried out using a family and population-based approach implemented in a commercial version of FImpute^19^, where no limitation was set for the reference population size. The FImpute has been proven to be able to provide accurate imputation with low computational requirements in dairy cattle^18,21^. In this study, the pedigree information was provided for all analyses. The pedigree was constructed by tracing back genotyped animals as many generations as possible, consisting of 6,102 bulls and 66,466 cows. The SNP-wise imputation accuracy was measured as correlation coefficient and concordance rate. The correlation was calculated as the Pearson correlation between observed and imputed genotypes (coded as 0, 1, or 2). The concordance rate was calculated as the proportion of correctly imputed genotypes to all imputed genotypes. In the present study, the imputation accuracy of preselected sequencing SNPs was investigated in both contemporary animals and old bulls.

#### Imputation of preselected sequencing SNPs for contemporary animals

In the present study, the customized LD chip was used as a reference panel for the imputation of DFS and FRA SNPs. The animals genotyped with the customerized LD chip were relatively young compared with animals genotyped with other versions of SNP chips. For the imputation of DFS and FRA SNPs, a random subset of animals genotyped with the customized LD chip were used as the target population, while all remaining animals genotyped with the customized LD chip were used as the reference population. Therefore, the target population for the imputation of DFS and FRA SNPs was the contemporary animals of the reference population. For contemporary animals, three imputation scenarios were compared regarding the imputation strategies of preselected sequencing SNPs. In scenario one, all genotyped individuals were imputed to the standard LD chip together with preselected DFS and FRA SNPs (LD + DFS + FRA). Here, for animals genotyped with the 54 K chip, only SNPs which appeared in the standard LD chip were kept for analyses. In scenario two, all genotyped individuals were directly imputed to 54 K together with the preselected DFS and FRA SNPs (one-step 54 K + DFS + FRA). In scenario three, individuals genotyped with different LD chips were firstly imputed to 54 K, and then individuals genotyped with 54 K or with imputed 54 K were further imputed to 54 K together with preselected DFS and FRA SNPs (two-step 54 K + DFS + FRA).

In all three scenarios, the imputation of DFS and FRA SNPs was assessed by masking genotypes of DFS and FRA SNPs for a random subset of 2,837 individuals (around 20%) from 14,372 individuals genotyped with the customized LD chip. Besides, for scenarios one-step and two-step 54 K + DFS + FRA, the imputation from the standard LD chip to the 54 K chip was assessed by masking genotypes for a random subset of 986 individuals (around 20%) from 4,913 individuals genotyped with the 54 K chip to become the standard LD chip. The accuracy of imputation was calculated by comparing the imputed genotypes with the observed genotypes for the masked SNPs. The proportion of animals being masked to assess the imputation accuracy was subjective, but a ratio about 80:20 is often used for dividing a whole dataset into training and test datasets. In the present study, considering the number of individuals being available for each SNP group, 20% animals were masked in order to have a good balance between validation and reference population sizes.

#### Imputation of preselected sequencing SNPs for old bulls

In the present study, most old bulls were genotyped with the 54 K chip and therefore lack of the information of DFS + FRA SNPs. To mimic the imputation of preselected sequencing SNPs (DFS + FRA SNPs) in old bulls, 500 SNPs selected from the standard LD chip were taken as preselected sequencing SNPs. Regarding the number of SNPs being selected to validate the imputation accuracy of preselected sequencing SNPs (500 in the present study), it was based on the considerations that the number of SNPs could be sufficient to control the sampling error in calculating imputation accuracy and moving this number of SNPs could not break the linkage disequilibrium structures among the remaining SNPs in the standard LD chip. Here, the reference population for the imputation of these 500 SNPs in old bulls comprised all genotyped cows. In total, 864 bulls were sires, grandsires, or great-grandsires of the reference population, while the remaining 4,157 bulls had more distant relationship with the reference population. To further investigate the influence of the number of genotyped progenies on the imputation of preselected sequencing SNPs in old bulls, the imputation accuracy average over these 500 SNPs was calculated for individuals with different number of genotyped daughters.

The distributions of MAF for SNPs in the standard LD chip were different from those of the preselected sequencing SNPs, which could potentially influence the assessment. Thus, we applied two strategies to select these 500 SNPs from the standard LD chip. One strategy was to randomly select 500 SNPs from the standard LD chip without considering MAF (Random500). In this case, the distribution of MAF for the 500 SNPs was similar to that of the standard LD chip. The other strategy was to select SNPs based on the MAF distribution of preselected sequencing SNPs (MAF500), where the number of SNPs being selected for each MAF class was equal to 500 multiplied the proportion of preselected sequencing SNPs in that MAF class (0.01–0.05, 0.05–0.10, 0.10–0.20, 0.20–0.30, 0.30–0.40, and 0.40–0.50). In this case, the distribution of MAF of these 500 selected SNPs was similar to that of preselected sequencing SNPs (DFS + FRA SNPs). Then, a two-step imputation was used to impute the selected 500 SNPs in old bulls. Animals genotyped with different LD chips were firstly imputed to the reduced 54 K (54 K minus the 500 SNPs), and then to the full 54 K (including the 500 SNPs). We repeated these procedures five times.

## Results

### Imputation of preselected sequencing SNPs for contemporary animals

For contemporary animals, imputation accuracy for DFS SNPs, FRA SNPs, and for the imputation from the standard LD chip to the 54 K chip are presented in Table 1. For different imputation scenarios, ranks of imputation accuracy were consistent regarding correlations and concordance rates. Among different SNP sets, imputation accuracy was highest for the imputation from the standard LD chip to the 54 K chip and lowest for FRA SNPs. For example, for SNPs with MAF higher than 0.01 in the two-step imputation to 54 K + DFS + FRA, average correlations ranged from 88.0% for FRA SNPs to 96.2% for the imputation from the standard LD chip to the 54 K chip, and average concordance rates ranged from 94.2% for FRA SNPs to 98.0% for the imputation from the standard LD chip to the 54 K chip. For different imputation scenarios, the highest accuracy was obtained using two-step 54 K + DFS + FRA and the lowest using one-step 54 K + DFS + FRA. For example, for DFS and FRA SNPs with MAF higher than 0.01, average correlations ranged from 88.2% when using one-step 54 K + DFS + FRA to 89.2% when using two-step 54 K + DFS + FRA, while average concordance rates ranged from 94.7% when using one-step 54 K + DFS + FRA to 95.1% when using two-step 54 K + DFS + FRA.

**Table 1:** Number of SNPs to be imputed and imputation accuracy (%) for contemporary animals.

SNP^**a**^	N	MAF^**b**^	Correlation			Concordance rate		
			LD + DFS + FRA^c^	One-step 54 K + DFS + FRA^d^	Two-step 54 K + DFS + FRA^e^	LD + DFS + FRA	One-step 54 K + DFS + FRA	Two-step 54 K + DFS + FRA
54 K	40,260	>0.00	—	94.4	95.4	—	98.0	98.3
	34,342	> 0.01	—	95.4	96.2	—	97.7	98.0
	30,494	> 0.05	—	95.9	96.6	—	97.5	97.9
DFS	1,681	>0.00	89.3	89.1	89.5	97.6	97.4	97.6
	1,329	> 0.01	91.4	90.8	91.8	97.0	96.8	97.0
	1,215	> 0.05	94.0	93.4	94.3	96.9	96.7	97.0
FRA	4,144	>0.00	86.1	85.0	86.5	95.9	95.7	96.0
	2,837	> 0.01	87.3	87.0	88.0	94.0	93.7	94.2
	2,469	> 0.05	89.0	88.7	89.5	93.5	93.2	93.8

^a^54K = imputation from the standard LD chip to 54 K; DFS SNPs = SNPs selected from major dairy breeds in Denmark-Finland-Sweden; FRA SNPs = SNPs selected from major dairy breeds in France.

^b^MAF = minor allele frequency.

^c^LD + DFS + FRA = imputation to standard LD chip together with preselected DFS and FRA SNPs.

^d^One-step 54 K + DFS + FRA = directly imputed to 54 K together with preselected DFS and FRA SNPs.

^e^Two-step 54 K + DFS + FRA = first imputed to 54 K and then to 54 K together with preselected DFS and FRA SNPs.

**Figure 1:**
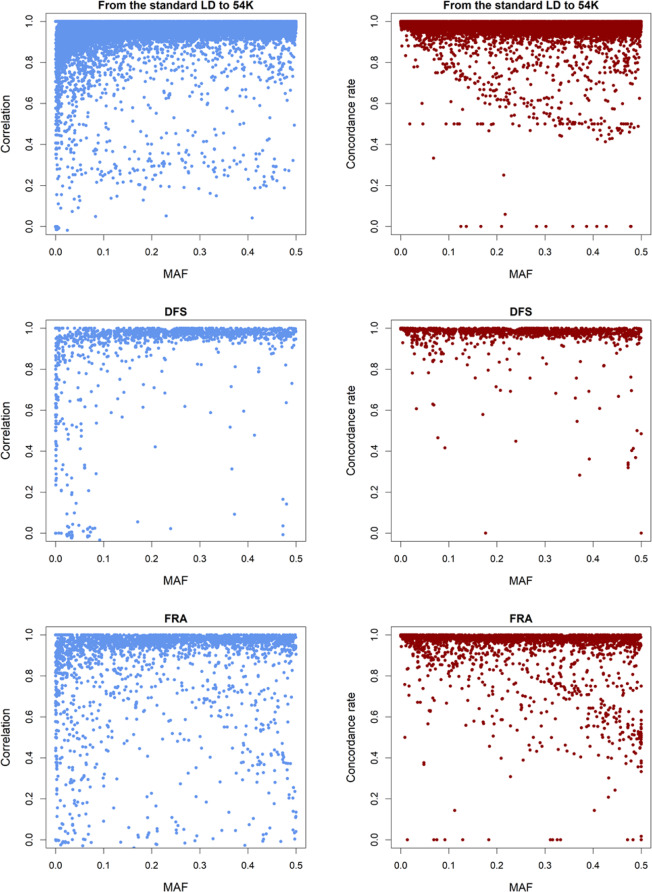
The accuracy of imputation using the two-step imputation in relation to MAF, for imputation from the standard LD chip to 54 K, and for imputation of DFS SNPs^2^ and FRA SNPs^3^ in contemporary animals.

The pattern of imputation accuracy in relation to MAF is presented in Table 1 and Fig. 1. Generally, SNPs with lower MAF tended to have lower correlations but higher concordance rates, which were observed within all three SNP sets. For example, in the scenario two-step 54 K + DFS + FRA, compared with no restriction on MAF, removing SNPs with MAF lower than 0.01 increased correlations by 0.8, 2.3, and 2.3 percentage points while decreased concordance rates by 0.3, 0.6, and 1.8 percentage points, for the imputation from the standard LD chip to the 54 K chip, the imputation of DFS SNPs, and the imputation of FRA SNPs, respectively.

### Imputation of preselected sequencing SNPs for old bulls

The proportions of SNPs in different MAF groups for SNPs in the standard LD chip, and DFS + FRA SNPs are presented in Table 2. Generally, more DFS and FRA SNPs had low MAF than SNPs in the standard LD chip. For example, the number of SNPs with MAF within 0.01~0.05 in DFS and FRA SNPs was more than twice as that in the standard LD chip. The distributions of MAF for the 500 selected SNPs in Random500 and MAF500 were similar to those for SNPs in the standard LD chip and DFS + FRA SNPs, respectively.

**Table 2:** Percentages of SNPs (%) in different MAF^a^ groups for SNPs in a standard LD chip or DFS SNPs plus FRA SNPs^b^.

MAF	Standard LD chip	DFS + FRA
0.01–0.05	4	9
0.05–0.10	6	10
0.10–0.20	15	22
0.20–0.30	21	22
0.30–0.40	26	18
0.40–0.50	28	19

^a^MAF = minor allele frequency.

^b^DFS SNPs plus FRA SNPs = SNPs selected from major dairy breeds in Denmark-Finland-Sweden and France.

In old bulls, imputation accuracy for MAF500 and Random500 are presented in Table 3. All results presented here were specific for SNPs with MAF higher than 0.01. The imputation accuracy for MAF500 and Random500 was similar and relatively high. For example, the correlation was 97.3% for MAF500 and 97.7% for Random500. In all cases, imputation accuracy of the 500 SNPs, which was used to mimic the imputation accuracy of preselected sequencing SNPs in old bulls, was higher than that of the preselected sequencing SNPs in contemporary animals.

**Table 3:** Imputation accuracy (%) for 500 SNPs randomly removed from the standard LD chip in bulls.

Scenario	No. of bulls	No. of daughters	Correlation	Concordance rate
MAF500^a^	5,021	—	97.3 (0.3)	98.6 (0.1)
	4,764	0	97.2 (0.2)	98.5 (0.1)
	144	>10	98.5 (0.2)	99.6 (0.0)
	45	>50	99.2 (0.2)	99.8 (0.0)
	26	>100	99.7 (0.1)	99.9 (0.0)
	16	>200	99.8 (0.1)	99.9 (0.0)
Random500^b^	5,021	—	97.7 (0.1)	98.4 (0.0)
	4,764	0	97.6 (0.1)	98.4 (0.1)
	144	>10	99.1 (0.2)	99.7 (0.0)
	45	>50	99.5 (0.1)	99.8 (0.0)
	26	>100	99.8 (0.0)	99.9 (0.0)
	16	>200	99.9 (0.0)	99.9 (0.0)

^a^MAF500 = randomly removed 500 SNPs from the standard LD chip according to minor allele frequency.

^b^Random500 = randomly removed 500 SNPs from the standard LD chip without considering minor allele frequency.

All genotyped cows were from 588 paternal half-sib families (sire families), with family size up to 2,182 in the largest half-sib family. Among these 588 sires of genotyped cows, 257 were genotyped. Imputation accuracy for bulls with a different number of genotyped daughters in MAF500 and Random500 are presented in Table 3. Compared with the imputation for contemporary animals, imputation accuracy was high even for bulls without genotyped daughters, e.g., correlations higher than 97.2% and concordance rates higher than 98.4%. Generally, imputation accuracy in terms of both correlation and concordance rate increased when bulls had more genotyped daughters, but the increase slowed down after bulls had more than 100 genotyped daughters. For example, for the scenario MAF500, correlation increased 2.5 percentage point on average when the number of daughters increased from 0 to 100, but only increased 0.1 percentage point on average when the number of daughters increased from 100 to 200.

## Discussion

The benefits of integrating sequencing SNPs preselected from association analyses and bioinformatics analyses have been well documented in genomic prediction^5–7^. However, the magnitude of improvement in prediction reliability could be highly related to the imputation accuracy of these preselected sequencing SNPs^25,26^. By using a customized LD chip as a reference panel, the present study investigated the imputation accuracy of sequencing SNPs preselected from association analyses and bioinformatics analyses in both contemporary animals and old bulls.

In contemporary animals, imputation accuracy for preselected sequencing SNPs was much lower than that for the imputation from the standard LD chip to the 54 K chip, although the number of animals used as the reference population for imputing preselected sequencing SNPs was almost three times as that for the imputation from the standard LD chip to the 54 K chip. The accurate imputation from the standard LD chip to the 54 K chip has been well documented in various dairy cattle populations^11,14^. One of the reasons for the high accuracy of imputation from the standard LD chip to the 54 K chip is that the standard LD chip was explicitly designed for the imputation to the 54 K chip, where both the SNPs in the standard LD chip and the SNPs to be imputed had relatively high MAF^11^. The preselected sequencing SNPs, however, had lower MAF than SNPs in the standard SNP chip. In the present study, 14.7% of SNPs from the 54 K chip had MAF lower than 0.1, whereas 20.9% of DFS SNPs and 30.8% of FRA SNPs had MAF lower than 0.1. The poor imputation accuracy of SNPs with MAF lower than 0.1 has been widely observed in previous studies^1,19,27^. Bolormaa *et al*.^28^ showed that missense SNPs had lower MAF and lower imputation accuracy than intergenic and intronic SNPs in sheep. In addition, the difference in MAF distributions between SNPs in the standard LD chip and the preselected sequencing SNPs also indicated that there would be weak linkage disequilibrium between these two groups of SNPs. Previous studies have shown that the linkage disequilibrium between SNPs to be imputed and their closest SNPs could influence the imputation accuracy^19,20^. Besides, within preselected sequencing SNPs, imputation accuracy was lower for FRA SNPs compared with DFS SNPs. One explanation for this could be that FRA SNPs had lower MAF than DFS SNPs. In addition, in order to be significant from association analyses, the genotyping and imputation qualities of these SNPs are usually good^29^.

Among different imputation strategies investigated in contemporary animals of the reference population, differences in imputation accuracy were observed for preselected sequencing SNPs. The two-step 54 K + DFS + FRA yielded higher imputation accuracy for DFS + FRA SNPs than the one-step LD + DFS + FRA. In two-step 54 K + DFS + FRA, the first step was to impute from the standard LD chip to the 54 K chip, and the second step was to impute from imputed 54 K to 54 K + DFS + FRA. Given the high accuracy for the first step, the two-step 54 K + DFS + FRA scenario could be considered as imputation from 54 K to 54 K + DFS + FRA. Regarding the imputation of DFS + FRA SNPs, the two-step 54 K + DFS + FRA scenario had a denser reference panel than the LD + DFS + FRA scenario, and therefore, a higher imputation accuracy. When imputing to 54 K + DFS + FRA, the two-step strategy yielded higher imputation accuracy than the one-step strategy which imputed missing genotypes of animals genotyped with the standard LD, the customized LD, and the 54 K chips simultaneously. The performance of a two-step imputation largely impacted by the performance of its first step^30^. In the two-step imputation used in the present study, the first step (imputation from the standard LD chip to the 54 K chip) achieved high imputation accuracy in terms of both correlation and concordance rates (higher than 95%), which laid a good foundation for the second step (from imputed 54 K to 54 K + DFS + FRA). The result was consistent with the previous studies. In Montbéliarde cows^17^, a similar two-step imputation strategy was used to impute preselected sequencing SNPs when using a customized low-density SNP chip as a reference panel. In other dairy cattle populations, a multiple-step imputation strategy was superior to a one-step imputation strategy when imputing from 3 K and 6 K chips to the HD chip where animals were firstly imputed to the 54 K chip and then to the HD chip^31^, or from the 54 K chip to WGS where animals were firstly imputed to the HD chip and then to WGS^32^. Besides, when using the one-step imputation strategy, the one-step LD + DFS + FRA slightly outperformed the one-step 54 K + DFS + FRA for imputing genotypes of DFS + FRA SNPs. A previous study in Holstein Friesian cattle showed that the imputation from the 54 K chip to WGS, where one half of the reference animals were genotyped with WGS and the remaining half were genotyped with the HD chip, outperformed that using all reference animals with WGS^32^. The authors of that study explained that the imputation algorithm cannot select the correct haplotype when there were multiple possible matches between sequencing haplotypes and the 54 K haplotypes, whereas less possible matches when the HD chip was added in between^32^. In the present study, more haplotypes could be phased in the data set of the one-step imputation to 54 K + DFS + FRA than that to LD + DFS + FRA. Due to a larger number of missing SNPs to be imputed, the difficulty to match to the correct haplotype when there were multiple matches available could be one of the reasons why one-step 54 K + DFS + FRA was slightly inferior to one-step LD + DFS + FRA regarding the imputation of DFS + FRA SNPs.

In the present study, both correlation and concordance rates were applied to measure the imputation accuracy. For contemporary animals, SNPs with lower MAF tended to have lower correlations but higher concordance rates. This was consistent with that reported in Ma *et al*.^18^. The reason was that the SNP with a lower MAF tended to be imputed to the major allele, and thus, a relatively high concordance rate could be achieved since most major alleles can be correctly imputed^18^. Another study performed by Mulder *et al*.^33^ showed that the concordance rate was highly influenced by the MAF, while the correlation was expected to be linearly related to the accuracy of genomic prediction. Calus *et al*.^20^ strongly suggested to use correlation as a measure of imputation accuracy, especially for sequencing SNPs with low MAF.

A previous study in Nordic Holsteins, however, reported a very limited improvement of reliability from integrating imputed sequencing SNPs with low MAF into genomic prediction^25^, which could be caused by the poor imputation of low MAF SNPs. The preselected sequencing SNPs generally had lower MAF than SNPs in the standard SNP chip (Table 2), more efforts on the imputation of sequencing SNPs with low MAF could be necessary in order to benefit from preselected sequencing SNPs. Or at least, filtering SNPs by imputation accuracy should be performed before further processes in genomic prediction or association studies^27^. In a previous study of Danish Jersey^7^, reliabilities of genomic prediction increased for milk (e.g. from 0.320 for 54 K to 0.424 for 54 K + DFS + FRA in Danish Jersey bulls) and protein (e.g. from 0.268 for 54 K to 0.306 for 54 K + DFS + FRA in Danish Jersey bulls) after integrating imputed DFS and FRA SNPs with imputation accuracy, measured by both correlation and concordance rate, higher than 0.8. Alternatively, dosage scores instead of integer genotype codes could be used as explanatory variables in genomic prediction and association studies in order to account for the uncertainty of imputation, especially when SNPs has low imputation accuracy^26,27^.

Previous studies showed that the closer the relationship between reference and target populations, the higher the imputation accuracy^12,34^. In the present study, the imputation for preselected sequencing SNPs in old bulls was investigated by imputing 500 SNPs of the standard LD chip. The imputation accuracies of these 500 selected SNPs, even for bulls without daughters, were higher than those for DFS and FRA SNPs in contemporary animals of the reference population. For old bulls, the missing genotypes of these 500 SNPs could be accurately imputed since most haplotypes of old bulls were available in genotyped cows, especially when old bulls had daughters, granddaughters, or great-granddaughters in the reference population. Furthermore, for SNPs with low MAF, both correlations and concordance rates in old bulls were quite high. These results indicated that the genotypes of old bulls could be accurately imputed by using information from multiple genotyped progenies. Thus, we suggested that it would be unnecessary to re-genotype old bulls for preselected sequencing SNPs with the customized LD chip.

In the present study, a customized LD chip was used as a reference panel to impute preselected sequencing SNPs. An alternative strategy is to impute all WGS SNPs using the WGS data as a reference panel. The reference population could be a small number of key animals selected from pedigree relationships or haplotype diversities or WGS data from relevant consortium such as the 1000 genomes bull project^35^. Brøndum *et al*.^16^ showed that the correlation between imputed and observed genotypes from the HD chip to WGS was 0.89 for Danish Jersey with a combined reference population. Compared with imputing preselected sequencing SNPs using a customized SNP chip as a reference panel, using the WGS data as a reference panel would be challenged by the limited reference population size, the intensive computational burden, and the difference in diversity and frequency of phased haplotypes between reference and target populations if only a small number of animals being sequenced^36^. The present study showed that the imputation accuracy using a customized LD chip as a reference panel was much higher than that based on WGS imputation reported in previous studies^1,16,32^. However, the imputation accuracy for preselected sequencing SNPs in contemporary animals was still not very high (correlation lower than 92% for SNPs with MAF higher than 0.01). It would be a good strategy to directly genotype these preselected sequencing SNPs, such as integrating the preselected sequencing SNPs into a standard LD chip or a standard 54 K chip.

## Conclusions

For contemporary animals, the imputation first to the 54 K chip and then to 54 K + DFS + FRA SNPs achieved highest imputation accuracy and SNPs with lower MAF tended to have lower correlations but higher concordance rates. Given the limited imputation accuracy especially for SNPs with low MAF, it would be a good strategy to directly genotype the preselected sequencing SNPs using a customized SNP chip. For old bulls, high imputation accuracy was achieved for SNPs with all MAF ranges, and therefore it would be unnecessary to re-genotype preselected sequencing SNPs.

## Data Availability

The datasets used in the present study are available from the corresponding author on reasonable request.
